# Uncovering Potential Therapeutic Targets in Colorectal Cancer by Deciphering Mutational Status and Expression of Druggable Oncogenes

**DOI:** 10.3390/cancers11070983

**Published:** 2019-07-14

**Authors:** Otília Menyhart, Tatsuhiko Kakisaka, Lőrinc Sándor Pongor, Hiroyuki Uetake, Ajay Goel, Balázs Győrffy

**Affiliations:** 1 2nd Department of Pediatrics, Semmelweis University, Tűzoltó utca 7-9, H-1094 Budapest, Hungary; 2 MTA TTK Lendület Cancer Biomarker Research Group, Institute of Enzymology, Hungarian Academy of Sciences, Magyar tudósok körútja 2., H-1117 Budapest, Hungary; 3 Center for Gastrointestinal Research & Center for Translational Genomics and Oncology, Baylor Scott & White Research Institute and Charles A. Sammons Cancer Center, 3410 Worth Street, Suite 610, Dallas, TX 75246, USA; 4 Department of Molecular Diagnostics, Therapeutics and Translational Oncology, Beckman Research Institute at City of Hope Comprehensive Cancer Center, 1218 S. Fifth Avenue, Monrovia, Suite 2226, CA 91016, USA; 5 Department of Specialized Surgeries, Graduate School of Medical and Dental Sciences, Tokyo Medical and Dental University, Tokyo 113-8519, Japan

**Keywords:** colon cancer, disruptive mutations, oncogenes, molecular targeted therapy, survival

## Abstract

Background: Numerous driver mutations have been identified in colorectal cancer (CRC), but their relevance to the development of targeted therapies remains elusive. The secondary effects of pathogenic driver mutations on downstream signaling pathways offer a potential approach for the identification of therapeutic targets. We aimed to identify differentially expressed genes as potential drug targets linked to driver mutations. Methods: Somatic mutations and the gene expression data of 582 CRC patients were utilized, incorporating the mutational status of 39,916 and the expression levels of 20,500 genes. To uncover candidate targets, the expression levels of various genes in wild-type and mutant cases for the most frequent disruptive mutations were compared with a Mann–Whitney test. A survival analysis was performed in 2100 patients with transcriptomic gene expression data. Up-regulated genes associated with worse survival were filtered for potentially actionable targets. The most significant hits were validated in an independent set of 171 CRC patients. Results: Altogether, 426 disruptive mutation-associated upregulated genes were identified. Among these, 95 were linked to worse recurrence-free survival (RFS). Based on the druggability filter, 37 potentially actionable targets were revealed. We selected seven genes and validated their expression in 171 patient specimens. The best independently validated combinations were *DUSP4* (*p* = 2.6 × 10^−12^) in *ACVR2A* mutated (7.7%) patients; *BMP4* (*p* = 1.6 × 10^−04^) in *SOX9* mutated (8.1%) patients; *TRIB2* (*p* = 1.35 × 10^−14^) in *ACVR2A* mutated patients; *VSIG4* (*p* = 2.6 × 10^−05^) in *ANK3* mutated (7.6%) patients, and *DUSP4* (*p* = 7.1 × 10^−04^) in *AMER1* mutated (8.2%) patients. Conclusions: The results uncovered potentially druggable genes in colorectal cancer. The identified mutations could enable future patient stratification for targeted therapy.

## 1. Introduction

Colorectal cancer (CRC) is a major cause of cancer-related deaths worldwide [1], with steeply increasing rates in some developed countries [2], especially among adults aged <50 years [3]. About 25% of patients are diagnosed with a metastatic disease and another 25% eventually develop metastases. CRC is molecularly heterogeneous, with significantly varying response to therapeutic intervention leading to poor prognosis among the majority of patients. In the early 2000s, treatment options, such as traditional chemotherapy (primarily based on oxaliplatin, fluoropyrimidine, and irinotecan) were complemented with more targeted approaches, such as the VEGF-inhibitor bevacizumab [4] and the EGFR-targeting monoclonal antibodies, cetuximab and panitumumab [5,6]. These therapeutic options resulted in an increased median survival of up to 30 months in the metastatic setting. Newer therapies for metastatic CRC focus mainly on angiogenesis inhibition: ziv-aflibercept as a second line treatment combined with FOLFIRI targets VEGF-A, VEGF-B, and PlGF [7], and the oral multi-kinase inhibitor regorafenib, which obstructs multiple tumor pathways and targets VEGFR1-3, TIE2, PDGFR, FGFR1, FGFR2, KIT and RET [8]. The recombinant fusion protein ramucirumab was approved in 2015 in combination with FOLFIRI, and inhibits ligand-stimulated activation of VEGFR2 receptors [9]. While many newer and developmental drugs such as these have multiple targets, the downstream signaling pathways are not yet well characterized, hindering effective biomarker development for patient stratification.

Recent efforts delivered CRC classifications using different molecular markers for patient stratification [10]. Advances in molecular technology are helping to identify critical driver genomic events associated with therapeutic response [11]. About 15% of tumors harbor microsatellite instability (MSI) as a consequence of defective DNA Mismatch Repair (MMR) genes, and these tumors are highly immunogenic. Cells with MMR defects are mutation-prone, particularly at microsatellite sequences, with 10-times more somatic mutations compared to non-MMR deficient tumors [12]. For metastatic dMMR/MSI-high disease, various immunotherapies (nivolumab, pembrolizumab and a combination of ipilimumab and nivolumab [13]) have been available since 2017. Nevertheless, the majority of CRCs are poorly immunogenic [14], and the evolution of targeted agents in CRC remains slow, mainly focusing on a selection of patients for anti-EGFR therapy [11]. Numerous biomarkers that confer innate resistance toward such agents have been described (e.g., mutations of *KRAS* and *NRAS*). Anti-EGFR therapy is more likely to be effective in KRAS wild-type patients who also harbor wild types *NRAS*, *BRAF* and *PIK3CA*, but this constellation appears only in up to 30% of the CRC patient population [15]. Overall, the low prevalence of some molecular events limits their applicability as disease biomarkers [11]. The emergence of positive predictive biomarkers for alternative treatment selection (e.g., *BRAF*^V600E^ and *RNF43* mutations, *ALK* and *NTRK1* fusions) is also slow, mostly with limited response rates [11]; therefore the identification of actionable genetic targets is highly sought after.

CRC arises through a multi-step process of sequential accumulation of molecular alterations. About five to seven oncogenic mutations are required for a malignant transformation [16], though according to some studies, three driver events are sufficient for tumorigenesis [12]. There has been a significant progress in identifying recurrent mutations over the past decades, and numerous oncogenes and tumor suppressors (e.g., *EGFR*, *APC*, *TP53*, *PTEN*, *SMAD4*, *FBXW7*, *SOX9*, and *ACVR2*) have been described [17]; however, to better understand the clinical significance and the underlying molecular mechanisms of action, further research is required. Since mutations have mainly been investigated in the context of the efficacy of anti-EGFR-therapy [11] and for most of them, targeted agents do not exist, it is particularly important to investigate the downstream effects of driver events as potential drug targets or markers of therapeutic outcome.

Driver mutations not only influence the gene itself and the corresponding protein production, but also impact downstream signaling pathways. In fact, the secondary effects of mutations may have stronger prognostic relevance than the primary genetic alterations [18], thus they might provide a viable pipeline to select targets for clinical intervention. The primary aim of our study was to identify differentially express genes as potential druggable targets linked to driver mutations. We focused on the 10 most frequent disruptive mutations, identified significantly upregulated genes associated with poor survival outcome and filtered out actionable genes to provide clinically relevant targets for drug development. The expression of the top genes was validated in an independent clinical cohort.

## 2. Methods

### 2.1. Sequencing and Expression Database

Expression and mutation data were retrieved from the TCGA repository (https://portal.gdc.cancer.gov/). Mutations identified with the mutect2 algorithm were downloaded in VCF format. The variations were chosen depending on their mutect2 ‘PASS’ status—mutations with 40x the overall coverage and a minimum of 5 reads were selected. The remaining mutations were annotated using the *snpEff* [19] program based on the GRCh38 human genome version. In the database construction, only the canonical isoforms were selected. The mutations were categorized as any, coding, non-coding and disruptive mutations, based on the impact on the protein sequence.

**Figure 1:**
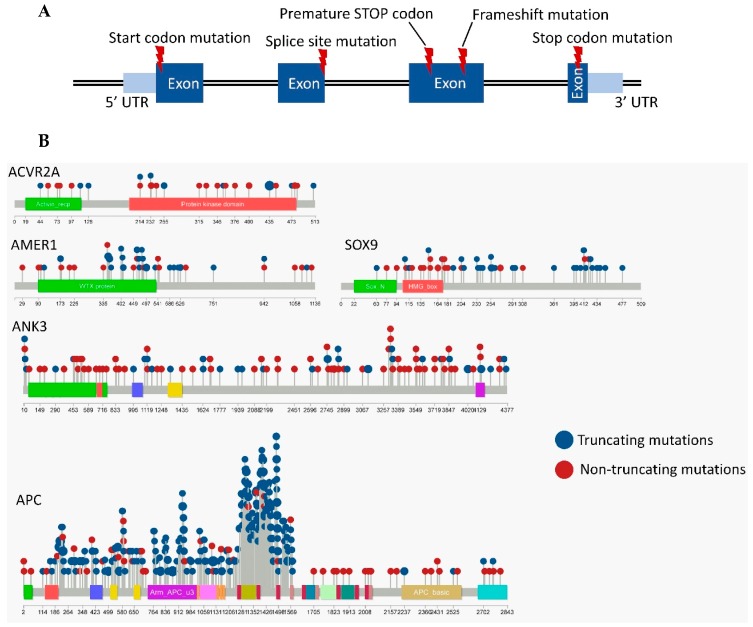
Disruptive mutations are defined as mutations that result in a disrupted protein structure. (**A**) These include mutations affecting the START or STOP codons, producing an early stop codon, affecting the splice-site, or resulting in a frame shift. (**B**) The locations of the mutations within the *ACVR2A*, *AMER1*, *SOX9*, *ANK3* and *APC* genes (larger circles represent recurrent mutations). Only mutations occurring in coding regions are included.

The disruptive mutations included those affecting the START or STOP codon, producing an early stop codon, affecting the splice-site (but not the splice region), or creating frame shifts (insertions and deletions affecting less than 3 nucleotides or exceeding the multiples of 3), as illustrated in Figure 1A [20].

The expression database was constructed from the raw HTseq-count read counts. The obtained read counts were normalized using the DESeq2 [21] algorithm.

### 2.2. Gene Selecting Algorithm

We split the specimens into two cohorts based on the mutation status. The normalized expression of the wild-type and mutant specimens was compared by a Mann–Whitney test. Only disruptive mutations were included, and we repeated the process for each selected gene. Statistical significance was accepted in case of *p* < 0.01 and a fold change (FC) cutoff at 1.44. The Benjamini–Hochberg false discovery rate was executed to safeguard against errors resulting from multiple hypothesis testing.

### 2.3. Survival Analysis

We searched PubMed Gene Expression Omnibus (GEO) data repository (https://www.ncbi.nlm.nih.gov/geo/) to identify colorectal cancer datasets with published survival times, as described previously [10]. The raw data were re-normalized, and the datasets were combined into a single database. To select the most reliable probe set for each gene, we used JetSet, a tool measuring and comparing different quality parameters for each probe set [22]. Survival analysis was performed for the selected genes using Cox proportional hazards regression. The analysis was performed using the auto-select best cutoff function, whereby all the possible cutoff values between the upper and lower quartiles were computed and the best performing threshold was used as a cutoff. Kaplan–Meier plots were drawn to visualize survival differences.

### 2.4. CRC Specimens for the Validation Cohort

For the clinical validation set up to identify top genes, we analyzed formalin-fixed paraffin-embedded (FFPE) cancer tissues from 171 CRC patients who underwent surgery at the Tokyo Medical and Dental University Hospital (TMDU), Tokyo, Japan from 2007 to 2011. All cases were histologically confirmed as adenocarcinoma and were classified into tumor-node-metastasis (TNM) stages after surgery according to the American Joint Committee on Cancer (AJCC), 7th edition. This study was conducted in accordance with the Declaration of Helsinki. All the procedures associated with this study were approved by the Institutional Review Boards of the Baylor Scott & White Research Institute (approval number: 003-180), and all the patients provided written informed consent.

### 2.5. Nucleic Acid Isolation and Gene Expression Analysis

The total RNA was isolated from the FFPE tissues using an ALL Prep DNA/RNA FFPE kit (Qiagen, Valencia, CA, USA). A synthesis of the complementary DNA (cDNA) was conducted with 500ng of the total RNA using the High Capacity cDNA Reverse Transcription Kit (Applied Biosystems, Foster City, CA, USA). qRT-PCR was performed using the SensiFAST™ SYBR^®^ Lo-ROX Kit (Bioline, London, UK) on the Quantstudio 7 Real Time PCR System (Applied Biosystems). The relative expression levels of the target genes were normalized against beta-actin using the 2^−ΔCT^ method. The PCR primers used in the current study are shown in Table S1.

### 2.6. Availability of Data and Material

Expression and mutation data were retrieved from the TCGA repository (https://portal.gdc.cancer.gov/).

## 3. Results

### 3.1. Database Setup

Data from 459 patients diagnosed with colon adenocarcinomas (COAD) and 170 patients with rectal adenocarcinomas (READ) were available from the TCGA repository, including a small subset of patients diagnosed with mucinous adenocarcinomas (COAD: 13.5%; READ: 7.6%). Most COAD patients were diagnosed with a clinical stage II disease, while roughly an equal number of READ patients were diagnosed with stage II and III disease. Altogether 55.4% of COAD patients were diagnosed in either stage I (16.6%) or stage II (38.8%), and about half (49.4%) of READ patients were diagnosed in stage I/II (stage I: 19.4%; stage II: 30.0%) (Table S2). The balanced incorporation of tumors with early and more advanced disease stages avoids selection bias for more aggressive tumors.

Venous invasion was present in about one fifth of all patients, and over one third of the patients were diagnosed with lymph node involvement. Males were slightly overrepresented in both COAD and READ. Over two third of patients were 60 years of age or older, with a median age of 68 years in COAD, and 66 years in READ. Residual disease was absent after surgery in most patients (COAD: 72.1%; READ: 74.7%). During the median follow-up period (COAD: 22.3 months; READ: 20.58 months), 22.2% of COAD and 15.9% of READ patients died. Only a small proportion (2.4%) of COAD patients and none of the READ patients were identified with MSI-high disease, although information about MSI was lacking for most patients (for details see Table S2). We combined the COAD and READ datasets when comparing mutation status and gene expression levels to increase the statistical power of the analysis.

### 3.2. Frequently Mutated Genes

**Figure 2:**
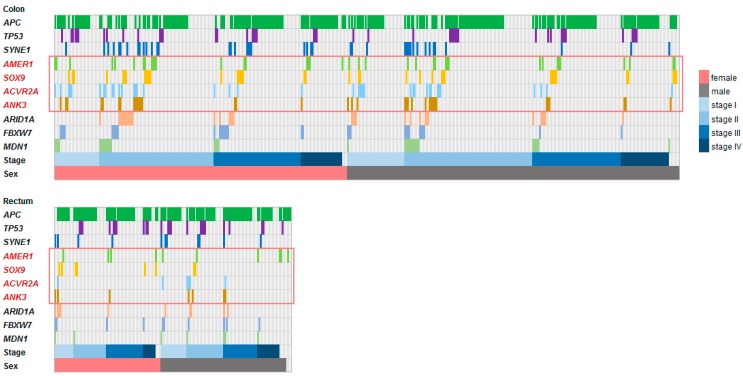
Distribution of the 10 most frequent disruptive mutations in the training cohort. Each column corresponds to a single patient. In some cases, multiple genes were co-mutated within the same tumor. Mutations are grouped based on sex (female—red, male—gray) and stage (shades of blue).

Altogether, 53,373 disruptive mutations were identified in the sample population affecting 14,021 out of 39,916 coding and non-coding genes, as annotated by *snpEff* (http://snpeff.sourceforge.net/index.html) [19]. The mutations displayed a skewed distribution: most mutations appeared in very few cases (1–2 specimens), while only 10 genes (excluding *TTN*) were mutated in more than 40 patients. The 40 most frequently mutated genes are listed in Table S3. We selected the top ten (excluding *TTN*) most frequently mutated genes (*APC*, *TP53*, *SYNE1*, *AMER1*, *SOX9*, *ACVR2A*, *ANK3*, *ARID1A*, *FBXW7*, *MDN1*) (Figure 2) for subsequent analysis.

The presence of selected disruptive mutations was not linked to patient sex in the entire dataset, nor to tumor stage in READ, although in COAD, two mutations remained differently distributed across tumor stages after multiple testing corrections: the *ACVR2A* mutations were linked to earlier tumor stages (stages I/II, *p* = 0.007) while the *APC* mutations were associated with an advanced disease (stage IV, *p* = 0.008). The locations of the disruptive mutations for the select genes are illustrated in Figure 1B. The raw data of Figure 1B and Figure 2 are presented in Tables S4 and S5. The expression levels of 20,500 genes were investigated in our patient population for correlation to each mutation.

### 3.3. Survival Analysis

For each mutation, the top 50 upregulated genes were selected for subsequent survival analysis, and all the genes were included when the number of upregulated genes was less than 50 (5 genes for *TP53* and 21 for *AMER1*). Out of the 426 upregulated genes, 95 were associated with worse outcome (Table S6), while 177 overexpressed genes correlated with better relapse-free survival (*p* < 0.05). For 117 genes, the association between gene expression and clinical outcome was not significant, and 38 genes could not be identified in the transcriptomic dataset.

### 3.4. Selection of Potentially Actionable Genes

**Figure 3:**
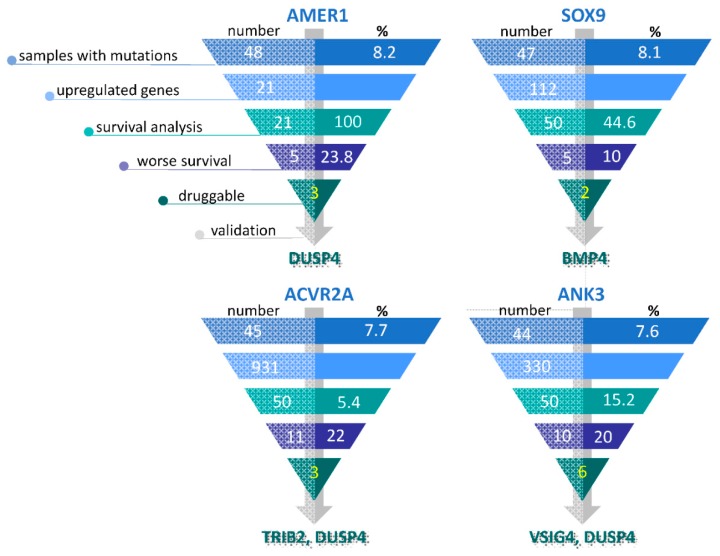
Overview of the gene selection process in tumor specimens containing *AMER1, SOX9, ACVR2A* and *ANK3* mutations. For each mutation, only the top 50 (or if less, all) upregulated genes were utilized in the subsequent survival analyses. The genes associated with worse survival were assessed for potential druggability, and the ultimately selected genes were validated in a clinical sample.

**Figure 4:**
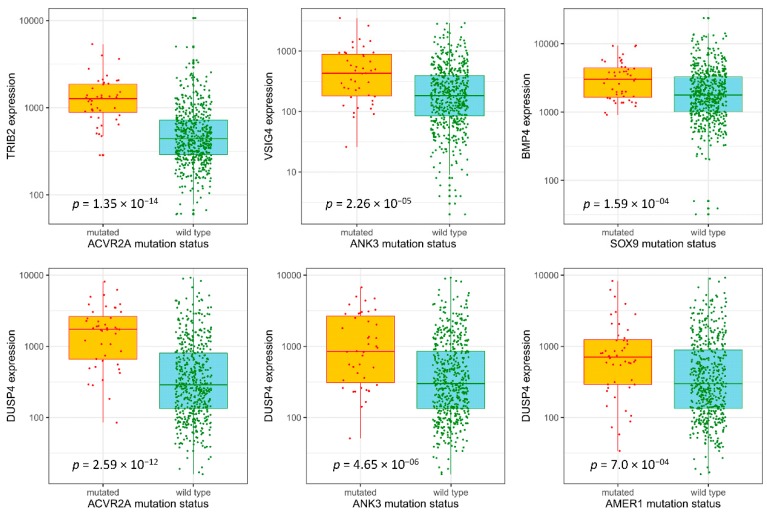
Differential expression of genes in mutant and wild-type tumors. mRNA levels of *TRIB2*, *DUSP4*, *VSIG4* and *BMP4* are significantly higher in *ACVR2A* (*TRIB2, DUSP4*), *ANK3* (*VSIG4, DUSP4*), *SOX9* (*BMP4*) and *AMER1* (*DUSP4*) mutant tumors compared to wild-type cases. The plots show Q1/Q2/Q3 within min-max range.

For potentially actionable genes, the Drug–Gene Interaction database DGIdb 3.0 [23] was analyzed. The database aggregates druggable gene categories from multiple sources and predicts druggability by various methods. Therefore, potentially actionable genes may or may not have existing drugs that target them. Out of the 95 upregulated genes linked to worst survival, 37 were classified as theoretically druggable (Table S6). We selected seven genes (*TRIB2*, *TRIM7*, *DUSP4*, *PKM2*, *VSIG4*, *BMP4* and *IL1RN*) based on MW *p*-values, expression differences, and effects on survival (Figures S1 and S2) for subsequent clinical validation. Out of the gene list, two upregulated genes (*TRIM7, DUSP4*) were linked to multiple mutations. The selection process of overexpressed genes linked to certain mutations is illustrated in Figure 3 and the differential expression for the top genes is presented in Figure 4.

### 3.5. Validation Sample Set

Altogether, 171 patient specimens were available for subsequent clinical validation of the selected genes. The majority of the patients were diagnosed with stage II and III disease, with 45 mm median tumor size. The median age at diagnosis was 67 years, although two third of the patient population was at least 60 years old, with slight overrepresentation of males. The majority of patients were diagnosed with the presence of venous invasion, and more than half with lymphatic invasion. The median recurrence-free survival (RFS) was 50 months, and 26.9% of patients recurred during follow up. The median overall survival (OS) was 60 months, and altogether, 19.9% of patients succumbed to the disease. Only 6.3% of patients were identified with microsatellite instability (MSI)-high disease, although information was unavailable for more than half of the patients. The characteristics of the validation dataset are summarized in Table 1.

### 3.6. Genes Associated with RFS in a Clinical Validation Set

**Figure 5:**
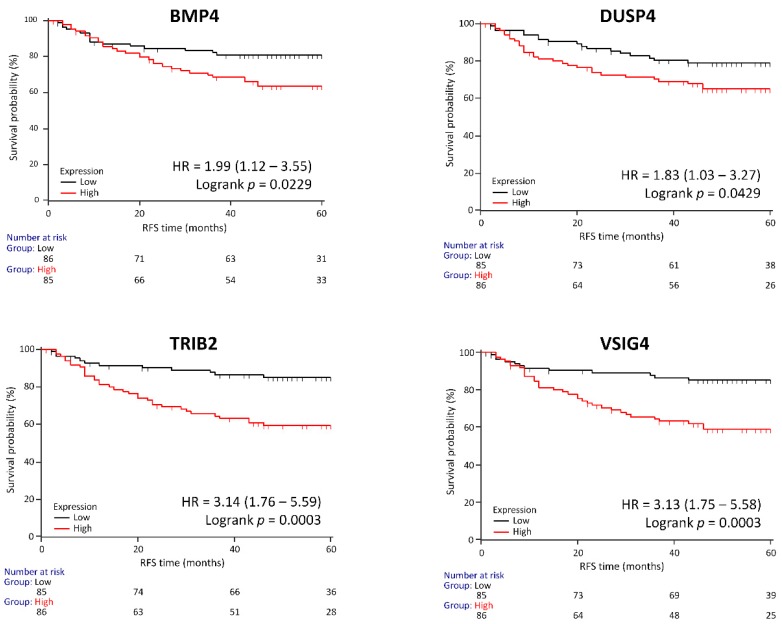
High expression of potentially actionable genes significantly associated with worse recurrence-free survival in the validation dataset.

The expression of the selected potentially actionable genes was validated with qPCR (Figure S3). High expression of *TRIB2* (*p* = 0.0003), VSIG4 (*p* = 0.0003), *BMP4* (*p* = 0.023) and *DUSP4* (*p* = 0.043) was associated with worse RFS in the validation dataset (Figure 5), although the expression of *TRIM7, PKM2* and *IL1RN* was not significantly different between patients with or without disease recurrence (*p* > 0.05). We used the median as a cutoff in the clinical validation cohort. We also analyzed RFS using Youden Index as a cutoff value (please see Figure S4). The genes associated with survival disadvantage were linked to mutations in *ACVR2A* (*TRIB2, DUSP4*), *ANK3* (*VSIG4, DUSP4*), *SOX9* (*BMP4*) and *AMER1* (*DUSP4*) (Figure 4).

## 4. Discussion

Despite the well-described driver genetic alterations in CRC, the availability of targeted therapies is limited, and the development of new personalized treatment methods has been rather slow. Mutations may impact downstream signaling pathways and alter the expression of potentially actionable genes. Evidence suggests that secondary genetic alterations may have a strong prognostic relevance [18], which provides an opportunity to utilize them in medical target selection. Following this approach, we identified four genes: *TRIB2*, *VSIG4*, *BMP4,* and *DUSP4.* These genes were overexpressed in CRC specimens with disruptive mutations, were linked to worse survival in multiple datasets and are deemed potentially actionable. The mutations affecting *ACVR2A, ANK3, SOX9* and *AMER1* may be utilized as biomarkers for patient stratification.

TRIB2 (Tribbles Pseudokinase 2) belongs to the Tribbles family of proteins that are structurally similar to kinases but lack catalytic activity. Growing evidence suggests the role of *TRIB2* as a pro-oncogene with a regulatory function underlying drug resistance both in solid and hematological malignancies [24,25]. In concordance with our findings, elevated TRIB2 expression was linked to poor prognosis in CRC, with higher expression in patients with frequent disease recurrence [26]. Consistently, ectopic or intrinsic high expression of TRIB2 in CRC confers resistance to chemotherapy by activating AKT [27]. TRIB2 down-regulation inhibits proliferation, induces cell-cycle arrest and senescence in CRC cell lines [26]. TRIB2 is a potential novel therapeutic target both for acute myeloid leukemia (AML) and acute lymphoblastic leukemia (ALL) [28]. Recent drug repurposing approaches identified the small-molecule protein kinase inhibitors afatinib and neratinib as active substances in AML cell lines that rapidly degrade TRIB2, eliciting survival benefits [29]. Afatinib has already been investigated both as a monotherapy and in combination regimens in CRC patients, but with limited antitumor activity [30], although biomarker-driven patient recruitment may increase therapy effectiveness. In our dataset, elevated *TRIB2* expression was specifically associated with the presence of *ACVR2A* mutations. *ACVR2A* (Activin A Receptor Type 2A) is one of the most frequently mutated genes in hypermutated CRC [31]. *ACVR2A* encodes a transmembrane receptor, which is a member of the TGF-beta superfamily of receptors with multiple biological functions, including the regulation of proliferation and cell migration [32]. ACVR2A down-regulation has been linked to advanced stage, lymphatic invasion and reduced survival, suggesting its prognostic significance in CRC [31]. Since *ACVR2A* mutations are most frequent in hypermutated CRCs, *TRIB2* may be most suitable as a therapeutic biomarker within this population, although the low proportion of patients with known MSI-high disease limits conclusions for MSI-specific biomarkers.

VSIG4 (V-Set and Immunoglobulin Domain Containing 4) is a newly identified immune checkpoint of the B7 family of immune regulatory proteins with similar functions as CTLA-4 and PD-1 in T-cell inhibition. Surface expression of VSIG4 protein is restricted to resting macrophages [33]. Its expression in tumor-associated macrophages is a potential facilitator of NSCLC development by inhibiting CD4+ and CD8+ T-cell proliferation and cytokine production [34]. In the current study, elevated *VSIG4* expression was associated with the presence of disruptive *ANK3* mutations and recurrent disease. ANK3 (Ankyrin G) provides cellular stability by anchoring cytoskeleton to the plasma membrane, and its down-regulation has been associated with poor prognosis [35] and increased invasive potential [36] in solid malignancies. Potential immune checkpoint inhibitor therapy may be most relevant for patients with upregulated VSIG4, especially those with simultaneous *ANK3* mutations.

Bone morphogenetic proteins (BMPs) have received much attention due to their role in tumor development and dissemination, and the wealth of conflicting studies indicates that BMPs may stimulate tumorigenesis in one cancer type but suppress it in another [37]. The *BMP4* (Bone Morphogenetic Protein 4) gene encodes a secreted ligand of the TGF-beta superfamily of proteins, resulting in the activation of SMAD family transcription factors [38]. BMP4 is overexpressed in CRC compared to normal tissue [39], and BMP4-overexpressing clones created from HCT116 cells exhibit enhanced migration and invasion [40]. In our data, *BMP4* upregulation was associated with the presence of *SOX9* mutations. *SOX9* (Sex-determining region Y (SRY)-box 9) is an intensely studied transcriptional factor, which plays critical roles in the development and lineage commitment of various tissues [41]. In the intestinal epithelium, it is mainly expressed in the progenitor cells of the colon and small intestines and plays a crucial role in stem-cell maintenance. It is also a downstream target of Wnt/β-catenin signaling, with possible roles in β-catenin regulation [42]. There is growing evidence of the importance of *SOX9* in CRC development. *SOX9* is mutated in 5–10% of all CRC cases [31] and leads to SOX9 overexpression [43]; this association is also confirmed in our dataset. It is likely a pro-oncogene, as high SOX9 expression has been linked to advanced tumor stage [44] and adverse prognosis in primary CRC [45]. BMP signaling and SOX9 interact in various developmental processes, such as chondrogenesis and the regulation of stem cells [46]. Targeting BMP4 may thus provide a viable therapeutic option, especially for patients with mesenchymal subtypes of CRC.

*DUSP4* (Dual Specificity Phosphatase 4) is a negative regulator of mitogenic signal transduction associated with proliferation and differentiation by dephosphorylating members of the MAPK superfamily (ERK1, ERK2, JNK) [47]. Higher DUSP4 expression characterizes MSI-high cell lines compared to microsatellite stable cells associated with increased cellular proliferation [48]. Consistently, in our dataset, significant *DUSP4* upregulation characterized tumors with various (*ACVR2A, AKN3* and *AMER1*) mutations, with the highest expression in *ACVR2A* mutant cases. Both *ACVR2A* and *ANK3* mutations have been mainly observed in hypermutated/MSI-high tumors [31,49], however the lack of information about MSI status for the majority of specimens does not allow conclusions for MSI-specific biomarkers. AMER1 (APC Membrane Recruitment Protein 1) is a tumor suppressor mutated in about 10% of CRC patients, especially in tumors with mesenchymal phenotypes linked to canonical Wnt-pathway inhibition [50]. Tumors lacking AMER1 mainly belong to the EMT subtype associated with poor prognosis and resistance to chemotherapy [50]. Thus, targeting *DUSP4* may provide a treatment approach for a wider selection of CRC patients with distinct molecular subtypes.

## 5. Conclusions

The evolution of precision medicine in CRC remains slow, with a limited efficacy of single-mutation/single-drug approaches [11]. Nevertheless, linking the mutation status of oncogenes with targetable opportunities in downstream signaling pathways provides a novel approach for therapy development. The proposed biomarkers represent divergent biological mechanisms and may be particularly suitable for distinct molecular subtypes of CRC. For example, *ACVR2A* mutations are most frequent in hypermutated CRC and therefore, targeting up-regulated *TRIB2* may potentially be suitable in such tumors, while *BMP4* may be a better suited biomarker for CRC patients with *SOX9* mutations and mesenchymal subtypes. In contrast, multiple mutations result in *DUSP4* upregulation, providing a potential treatment for a larger selection of patients. The suggested biomarker-defined patient stratification may provide an attractive approach toward therapy optimization.

## Figures and Tables

**Table 1:** Clinical characterization of the 171 CRC patients from the validation dataset.

Characteristic	(%)
**Sex**	
Male	55.6
Female	44.4
**Age/Years**	
<60	24.6
≥60	75.4
**Stage**	
I	9.9
II	41.5
III	41.5
IV	7.0
**Venous Invasion**	
Present	86.0
Absent	13.5
N/A	0.6
**Lymphatic Invasion**	
Present	57.9
Absent	41.5
N/A	0.6
**Microsatellite Instability**	
Yes	6.4
No	42.7
N/A	50.9
**Recurrence (Median Follow up 50 Months)**	26.9

N/A—data unavailable.

## Supplementary Materials

The following are available online at https://www.mdpi.com/2072-6694/11/7/983/s1: Figure S1: Sensitivity and specificity of the seven selected potentially actionable genes associated with frequent disruptive mutations by ROC analysis, Figure S2: High expression of the selected seven potentially actionable genes from the training dataset is linked to worse relapse-free survival based on Cox proportional hazard regressions at *p* < 0.05, Figure S3: Box plots indicating expression of the potentially actionable genes in the validation cohort, Figure S4: Recurrence-free survival of the clinical validation cohort, stratifying the population into high and low risk using the Youden index as a cutoff, Table S1: Primer sequences used in the qPCR validation, Table S2: Clinical characterization of colon and rectal cancer patient populations from the training datasets, Table S3: Top 40 most frequently mutated genes (disruptive mutations exclusively) in the CRC patient population (*n* = 582), Table S4: Mutations in the training dataset, Table S5: Raw data including stage, sex and mutations in the training dataset, Table S6: List of upregulated genes associated with worse clinical outcome (RFS, *p* < 0.05).

## Abbreviations

CRC	colorectal carcinoma
MSI	microsatellite instability
MMR	DNA Mismatch Repair
FFPE	formalin-fixed paraffin-embedded
TNM	tumor-node-metastasis
AJCC	American Joint Committee on Cancer
COAD	colon adenocarcinomas
READ	rectal adenocarcinomas
RFS	recurrence free survival
OS	overall survival
AML	acute myeloid leukemia
ALL	acute lymphoblastic leukemia
BMP	bone morphogenetic protein

## References

[1] Ferlay J., Colombet M., Soerjomataram I., Mathers C., Parkin D.M., Piñeros M., Znaor A., Bray F. (2019). Estimating the global cancer incidence and mortality in 2018: GLOBOCAN sources and methods. Int. J. Cancer.

[2] Menyhart O., Fekete J.T., Gyorffy B. (2018). Demographic shift disproportionately increases cancer burden in an aging nation: current and expected incidence and mortality in Hungary up to 2030. Clin. Epidemiol..

[3] Siegel R.L., Miller K.D., Fedewa S.A., Ahnen D.J., Meester R.G.S., Barzi A., Jemal A. (2017). Colorectal cancer statistics, 2017. CA Cancer J. Clin..

[4] Hurwitz H., Fehrenbacher L., Novotny W., Cartwright T., Hainsworth J., Heim W., Berlin J., Baron A., Griffing S., Holmgren E. (2004). Bevacizumab plus irinotecan, fluorouracil, and leucovorin for metastatic colorectal cancer. N. Engl. J. Med..

[5] Van Cutsem E., Kohne C.H., Hitre E., Zaluski J., Chang Chien C.R., Makhson A., D’Haens G., Pinter T., Lim R., Bodoky G. (2009). Cetuximab and chemotherapy as initial treatment for metastatic colorectal cancer. N. Engl. J. Med..

[6] Douillard J.Y., Siena S., Cassidy J., Tabernero J., Burkes R., Barugel M., Humblet Y., Bodoky G., Cunningham D., Jassem J. (2010). Randomized, phase III trial of panitumumab with infusional fluorouracil, leucovorin, and oxaliplatin (FOLFOX4) versus FOLFOX4 alone as first-line treatment in patients with previously untreated metastatic colorectal cancer: the PRIME study. J. Clin. Oncol..

[7] Van Cutsem E., Tabernero J., Lakomy R., Prenen H., Prausova J., Macarulla T., Ruff P., van Hazel G.A., Moiseyenko V., Ferry D. (2012). Addition of aflibercept to fluorouracil, leucovorin, and irinotecan improves survival in a phase III randomized trial in patients with metastatic colorectal cancer previously treated with an oxaliplatin-based regimen. J. Clin. Oncol..

[8] Grothey A., Van Cutsem E., Sobrero A., Siena S., Falcone A., Ychou M., Humblet Y., Bouche O., Mineur L., Barone C. (2013). Regorafenib monotherapy for previously treated metastatic colorectal cancer (CORRECT): an international, multicentre, randomised, placebo-controlled, phase 3 trial. Lancet.

[9] Tabernero J., Yoshino T., Cohn A.L., Obermannova R., Bodoky G., Garcia-Carbonero R., Ciuleanu T.E., Portnoy D.C., Van Cutsem E., Grothey A. (2015). Ramucirumab versus placebo in combination with second-line FOLFIRI in patients with metastatic colorectal carcinoma that progressed during or after first-line therapy with bevacizumab, oxaliplatin, and a fluoropyrimidine (RAISE): A randomised, double-blind, multicentre, phase 3 study. Lancet Oncol..

[10] Sztupinszki Z., Győrffy B. (2016). Colon cancer subtypes: concordance, effect on survival and selection of the most representative preclinical models. Sci. Rep..

[11] Dienstmann R., Vermeulen L., Guinney J., Kopetz S., Tejpar S., Tabernero J. (2017). Consensus molecular subtypes and the evolution of precision medicine in colorectal cancer. Nat. Rev. Cancer.

[12] Tomasetti C., Marchionni L., Nowak M.A., Parmigiani G., Vogelstein B. (2015). Only three driver gene mutations are required for the development of lung and colorectal cancers. Proc. Natl. Acad. Sci. USA.

[13] Overman M.J., Lonardi S., Wong K.Y.M., Lenz H.J., Gelsomino F., Aglietta M., Morse M.A., Cutsem E.V., McDermott R., Hill A. (2018). Durable Clinical benefit with nivolumab plus ipilimumab in dna mismatch repair–deficient/microsatellite instability–high metastatic colorectal cancer. J. Clin. Oncol..

[14] Becht E., de Reynies A., Giraldo N.A., Pilati C., Buttard B., Lacroix L., Selves J., Sautes-Fridman C., Laurent-Puig P., Fridman W.H. (2016). Immune and Stromal classification of colorectal cancer is associated with molecular subtypes and relevant for precision immunotherapy. Clin. Cancer Res..

[15] De Roock W., Claes B., Bernasconi D., De Schutter J., Biesmans B., Fountzilas G., Kalogeras K.T., Kotoula V., Papamichael D., Laurent-Puig P. (2010). Effects of KRAS, BRAF, NRAS, and PIK3CA mutations on the efficacy of cetuximab plus chemotherapy in chemotherapy-refractory metastatic colorectal cancer: A retrospective consortium analysis. Lancet Oncol..

[16] Calabrese P., Mecklin J.P., Järvinen H.J., Aaltonen L.A., Tavaré S., Shibata D. (2005). Numbers of mutations to different types of colorectal cancer. BMC Cancer.

[17] Fearon E.R. (2011). Molecular genetics of colorectal cancer. Annu. Rev. Pathol..

[18] Nagy Á., Pongor L.S., Szabó A., Santarpia M., Győrffy B. (2017). KRAS driven expression signature has prognostic power superior to mutation status in non-small cell lung cancer. Int. J. Cancer.

[19] Cingolani P., Platts A., Wang le L., Coon M., Nguyen T., Wang L., Land S.J., Lu X., Ruden D.M. (2012). A program for annotating and predicting the effects of single nucleotide polymorphisms, SnpEff: SNPs in the genome of Drosophila melanogaster strain w1118; iso-2; iso-3. Fly.

[20] Jay J.J., Brouwer C. (2016). Lollipops in the clinic: Information Dense mutation plots for precision medicine. PLoS ONE.

[21] Varet H., Brillet-Gueguen L., Coppee J.Y., Dillies M.A. (2016). SARTools: A DESeq2- and EdgeR-Based R Pipeline for comprehensive differential analysis of RNA-Seq Data. PLoS ONE.

[22] Li Q., Birkbak N.J., Gyorffy B., Szallasi Z., Eklund A.C. (2011). Jetset: selecting the optimal microarray probe set to represent a gene. BMC Bioinform..

[23] Cotto K.C., Wagner A.H., Feng Y.Y., Kiwala S., Coffman A.C., Spies G., Wollam A., Spies N.C., Griffith O.L., Griffith M. (2018). DGIdb 3.0: A redesign and expansion of the drug-gene interaction database. Nucleic Acids Res..

[24] Keeshan K., He Y., Wouters B.J., Shestova O., Xu L., Sai H., Rodriguez C.G., Maillard I., Tobias J.W., Valk P. (2006). Tribbles homolog 2 inactivates C/EBPalpha and causes acute myelogenous leukemia. Cancer Cell.

[25] Hill R., Kalathur R.K., Colaco L., Brandao R., Ugurel S., Futschik M., Link W. (2015). TRIB2 as a biomarker for diagnosis and progression of melanoma. Carcinogenesis.

[26] Hou Z., Guo K., Sun X., Hu F., Chen Q., Luo X., Wang G., Hu J., Sun L. (2018). TRIB2 functions as novel oncogene in colorectal cancer by blocking cellular senescence through AP4/p21 signaling. Mol. Cancer.

[27] Hill R., Madureira P.A., Ferreira B., Baptista I., Machado S., Colaco L., Dos Santos M., Liu N., Dopazo A., Ugurel S. (2017). TRIB2 confers resistance to anti-cancer therapy by activating the serine/threonine protein kinase AKT. Nat. Commun..

[28] Liang K.L., Rishi L., Keeshan K. (2013). Tribbles in acute leukemia. Blood.

[29] Foulkes D.M., Byrne D.P., Yeung W., Shrestha S., Bailey F.P., Ferries S., Eyers C.E., Keeshan K., Wells C., Drewry D.H. (2018). Covalent inhibitors of EGFR family protein kinases induce degradation of human Tribbles 2 (TRIB2) pseudokinase in cancer cells. Sci. Signal.

[30] De Pauw I., Wouters A., Van den Bossche J., Peeters M., Pauwels P., Deschoolmeester V., Vermorken J.B., Lardon F. (2016). Preclinical and clinical studies on afatinib in monotherapy and in combination regimens: Potential impact in colorectal cancer. Pharmacol. Ther..

[31] (2012). Comprehensive molecular characterization of human colon and rectal cancer. Nature.

[32] Bauer J., Ozden O., Akagi N., Carroll T., Principe D.R., Staudacher J.J., Spehlmann M.E., Eckmann L., Grippo P.J., Jung B. (2015). Activin and TGFbeta use diverging mitogenic signaling in advanced colon cancer. Mol. Cancer.

[33] Vogt L., Schmitz N., Kurrer M.O., Bauer M., Hinton H.I., Behnke S., Gatto D., Sebbel P., Beerli R.R., Sonderegger I. (2006). VSIG4, a B7 family-related protein, is a negative regulator of T cell activation. J. Clin. Investig..

[34] Liao Y., Guo S., Chen Y., Cao D., Xu H., Yang C., Fei L., Ni B., Ruan Z. (2014). VSIG4 expression on macrophages facilitates lung cancer development. Lab. Investig..

[35] Glinsky G.V., Berezovska O., Glinskii A.B. (2005). Microarray analysis identifies a death-from-cancer signature predicting therapy failure in patients with multiple types of cancer. J. Clin. Investig..

[36] Wang T., Abou-Ouf H., Hegazy S.A., Alshalalfa M., Stoletov K., Lewis J., Donnelly B., Bismar T.A. (2016). Ankyrin G expression is associated with androgen receptor stability, invasiveness, and lethal outcome in prostate cancer patients. J. Mol. Med..

[37] Bach D.H., Park H.J., Lee S.K. (2017). The dual role of bone morphogenetic proteins in cancer. Mol. Ther. Oncol..

[38] Bragdon B., Moseychuk O., Saldanha S., King D., Julian J., Nohe A. (2011). Bone morphogenetic proteins: A critical review. Cell. Signal..

[39] Irshad S., Bansal M., Guarnieri P., Davis H., Al Haj Zen A., Baran B., Pinna C.M.A., Rahman H., Biswas S., Bardella C. (2017). Bone morphogenetic protein and notch signalling crosstalk in poor-prognosis, mesenchymal-subtype colorectal cancer. J. Pathol..

[40] Deng H., Makizumi R., Ravikumar T.S., Dong H., Yang W., Yang W.L. (2007). Bone morphogenetic protein-4 is overexpressed in colonic adenocarcinomas and promotes migration and invasion of HCT116 cells. Exp. Cell Res..

[41] Wegner M. (2010). All purpose Sox: The many roles of Sox proteins in gene expression. Int. J. Biochem. Cell Biol..

[42] Blache P., van de Wetering M., Duluc I., Domon C., Berta P., Freund J.N., Clevers H., Jay P. (2004). SOX9 is an intestine crypt transcription factor, is regulated by the Wnt pathway, and represses the CDX2 and MUC2 genes. J. Cell Biol..

[43] Javier B.M., Yaeger R., Wang L., Sanchez-Vega F., Zehir A., Middha S., Sadowska J., Vakiani E., Shia J., Klimstra D. (2016). Recurrent, truncating SOX9 mutations are associated with SOX9 overexpression, KRAS mutation, and TP53 wild type status in colorectal carcinoma. Oncotarget.

[44] Matheu A., Collado M., Wise C., Manterola L., Cekaite L., Tye A.J., Canamero M., Bujanda L., Schedl A., Cheah K.S. (2012). Oncogenicity of the developmental transcription factor Sox9. Cancer Res..

[45] Lu B., Fang Y., Xu J., Wang L., Xu F., Xu E., Huang Q., Lai M. (2008). Analysis of SOX9 expression in colorectal cancer. Am. J. Clin. Pathol..

[46] Pritchett J., Athwal V., Roberts N., Hanley N.A., Hanley K.P. (2011). Understanding the role of SOX9 in acquired diseases: lessons from development. Trends Mol. Med..

[47] Keyse S.M. (2008). Dual-specificity MAP kinase phosphatases (MKPs) and cancer. Cancer Metastasis Rev..

[48] Groschl B., Bettstetter M., Giedl C., Woenckhaus M., Edmonston T., Hofstadter F., Dietmaier W. (2013). Expression of the MAP kinase phosphatase DUSP4 is associated with microsatellite instability in colorectal cancer (CRC) and causes increased cell proliferation. Int. J. Cancer.

[49] Yeon S.Y., Jo Y.S., Choi E.J., Kim M.S., Yoo N.J., Lee S.H. (2018). Frameshift Mutations in Repeat sequences of ANK3, HACD4, TCP10L, TP53BP1, MFN1, LCMT2, RNMT, TRMT6, METTL8 and METTL16 genes in colon cancers. Pathol. Oncol. Res..

[50] Sanz-Pamplona R., Lopez-Doriga A., Pare-Brunet L., Lazaro K., Bellido F., Alonso M.H., Ausso S., Guino E., Beltran S., Castro-Giner F. (2015). Exome sequencing reveals AMER1 as a frequently mutated gene in colorectal cancer. Clin. Cancer Res..

